# Frank’s Sign and Risk of Cardiovascular Events in Patients Hospitalized for Pneumonia: A Prospective Single-Center Cohort Study

**DOI:** 10.3390/jcm15103656

**Published:** 2026-05-09

**Authors:** Laure Cochand, Nils Bürgisser, Clement P Buclin, Aline Habegger, Jeremy H. Martin, Marion Hell, Solenne Perrenoud, Daniel Genné

**Affiliations:** 1 Division of General Internal Medicine, Department of Medicine, Biel Hospital, 2502 Biel, Switzerland; laure.cochand@szb-chb.ch (L.C.);; 2 Division of General Internal Medicine, Department of Medicine, Geneva University Hospitals, 1205 Geneva, Switzerland; 3 Faculty of Medicine, University of Geneva, 1211 Geneva, Switzerland

**Keywords:** cardiovascular diseases, pneumonia, cohort studies, risk factors, physical examination

## Abstract

**Background/Objectives:** Frank’s sign (FS), an acquired diagonal earlobe crease, has been associated with underlying cardiovascular (CV) disease. Pneumonia, a leading cause of hospitalization, may trigger CV events. FS may help identify patients at increased risk of CV complications following pneumonia. We aimed to evaluate the association between FS and CV events following pneumonia. **Methods**: We conducted a single-center prospective cohort study of internal medicine patients hospitalized with community-acquired pneumonia at a secondary hospital in Switzerland, followed for 18 months. FS was assessed at inclusion by trained investigators. Baseline characteristics were recorded. The primary outcome was a composite of CV endpoints: new-onset atrial fibrillation/flutter, myocardial infarction, hospitalization for heart failure, peripheral arterial ischemic events, or stroke. Events were identified through hospital records and general practitioner data between 2020 and 2023. **Results**: Among 203 patients (median age 76.0 years [IQR 65.0 to 83.0]; 41.4% female), 71.2% had FS. During follow-up, 103 CV events occurred. In univariate Cox analysis, FS was associated with an increased hazard of CV events (*p* = 0.042). After adjustment for confounders, FS was not significantly associated with CV events (HR 0.97; 95% CI 0.59–1.59). Only a history of CV disease and CV risk factors were associated with an increased hazard of subsequent cardiovascular events following pneumonia. **Conclusions**: In this population of patients hospitalized with pneumonia, Frank’s sign was not independently associated with cardiovascular events and appears to reflect underlying cardiovascular risk burden. It should not be interpreted as an independent prognostic marker.

## 1. Introduction

Pneumonia is a common infection that affects nearly 350 million people worldwide each year and remains a leading cause of mortality [1]. It places a significant strain on the healthcare system, with hospital admissions for respiratory diseases increasing by 37% compared with a 6.4% rise in admissions for other causes in the last decade [2]. Recent research has highlighted that pneumonia is not only a pulmonary condition but also a systemic disease that can precipitate cardiovascular events [3]. Moreover, the severity of the pneumonia appears to correlate with the risk of myocardial infarction [4]. Corrales-Medina et al. reported that within 30 days of pneumonia onset, the most frequent cardiac complications include heart failure (14%), arrhythmias (5%), and acute coronary syndrome or unstable angina (5%) [5]. Notably, these complications can arise even in patients without a prior history of cardiac disease. They are also associated with increased short- and long-term mortality, with the excess risk persisting for up to 10 years after the infection compared to other infections such as urinary tract infections [4].

In an era of medicine increasingly relying on non-clinical skills, such as laboratory testing and imaging, there is a growing need to strengthen skills in semiology. Strong clinical skills have been associated with a greater diagnostic accuracy in hospitalized patients [6]. This is particularly important as studies have shown that medical students often demonstrate limited proficiency in clinical examination [7]. One easily recognizable, yet relatively unknown, clinical sign is Frank’s sign.

Frank’s sign, an acquired prominent diagonal crease in the lobule portion of the auricula, was first described by Sanders T. Frank in 1973. Frank was the first to hypothesize a relationship between this sign and cardiovascular disease. In his original work, he found that out of 20 patients who showed Frank’s sign, 19 had one or more risk factors for coronary artery disease [8]. Since then, many studies have shown its association with coronary heart disease, cardiac morbidity and mortality [9]. Moreover, Frank’s sign has been associated with a range of cardiovascular and vascular conditions, including peripheral arterial disease, and cerebrovascular disease, supporting its role as a marker of systemic vascular pathology [10]. Autopsy-based studies have demonstrated that Frank’s sign is associated with more extensive systemic atherosclerosis, and that greater severity of the sign correlates with increased atherosclerotic burden and cardiovascular disease [9]. More recently, it has also been shown to be independently linked to higher cardiovascular risk scores [11].

Whilst the exact physiopathology of Frank’s sign remains unclear, several mechanisms have been proposed to explain this association. A first hypothesis suggests that involvement of small, distal arteries affects both the heart and the earlobe [9]. Another hypothesis argues that the earlobe’s particular sensitivity to hypoxia results from reduced vascularization linked to its embryologic development, a phenomenon that correlates with histopathological findings in the myocardium [12].

Whilst the association between Frank’s sign and cardiovascular event has been clearly established, there is a lack of literature about its uses as a predictor of cardiovascular event after community-acquired pneumonia.

In this prospective study, we aim to identify the prevalence of Frank’s sign in a cohort of internal medicine patients hospitalized with pneumonia, as well as to describe its association with future cardiovascular events. Our hypothesis is that Frank’s sign may help identify patients at increased risk of cardiovascular events following lower respiratory tract infection. We aim to evaluate the predictive value of an easily recognizable clinical sign for cardiovascular events, with the goal of enhancing overall patient care and improving prevention strategies.

## 2. Materials and Methods

### 2.1. Study Design and Setting

We conducted a prospective study in the internal medicine department of a public secondary hospital in Biel, canton of Bern, Switzerland, from January 2020 to January 2023. The department cared for a mean of 2781 inpatients annually during the study period.

### 2.2. Enrollement Procedure and Inclusion and Exclusion Criteria

Adult patients (≥18 years old) newly hospitalized in the internal medicine department with a diagnosis of pneumonia were screened by their physician and referred to the primary investigator.

Inclusion criteria:

- Age ≥ 18 years
- Hospitalization in the internal medicine department
- Diagnosis of pneumonia, defined by the presence of the following criteria:
  ○ New infiltrate on chest radiography;
  ○ Leukocytosis, leukopenia, or left shift with normal leukocyte count;
  ○ New clinical symptoms (e.g., cough, fever, dyspnea, or changes in sputum color, consistency or volume).

Exclusion criteria:

- Nosocomial pneumonia;
- Aspiration pneumonia;
- Inability or refusal to provide informed consent.

After inclusion, presence of the Frank’s sign was evaluated by the treating physicians with the help of one of the primary investigators if needed. Frank’s sign was defined as a diagonal earlobe crease extending from the tragus to the earlobe margin. All participating physicians received training to recognize Frank’s sign.

Based on this assessment, patients were then classified into two groups: the Frank’s sign group (patients with Frank’s sign) and the control group (patients without Frank’s sign). No distinction was made between different types of community-acquired pneumonia. Viral pneumonia that occurred during the course of the study, including SARS-CoV-2 pneumonia, was included.

**Figure 1:**
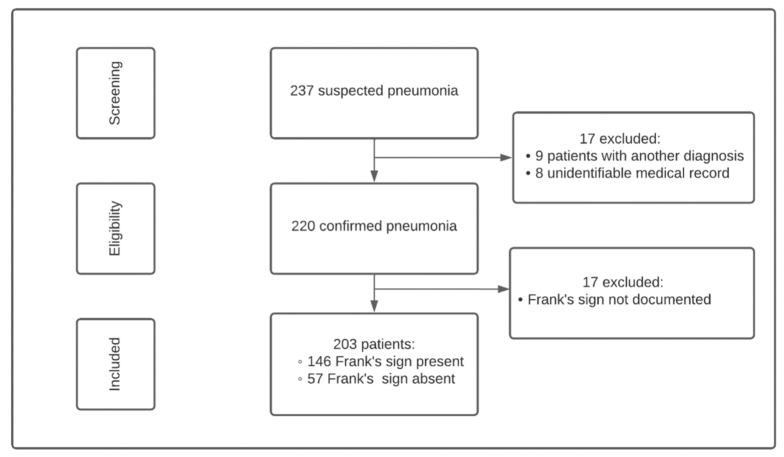
Flowchart leading to the final 203 patients included in the cohort.

The patient selection process is summarized in Figure 1. A total of 237 patients were reported for inclusion in the study, of whom 17 were excluded after review by the primary investigators. Nine patients were excluded for the absence of pneumonia, and eight were due to unidentifiable records. Of the 220 remaining patients, 17 were further excluded because the documentation of Frank’s sign was missing at the time of inclusion, resulting in 203 patients included in the final analysis.

### 2.3. Primary Outcome

The main goal of the study was to evaluate the incidence of cardiovascular events after a pneumonia in patient with Frank’s sign compared to patients without Frank’s sign. New cardiovascular events were defined as a composite of cardiovascular event as follows:

- New episode of atrial fibrillation or flutter;
- Myocardial infarction (STEMI or NSTEMI);
- Hospitalization for heart failure;
- Peripheral arterial ischemic event;
- Stroke.

The composite outcome was chosen to capture the overall burden of clinically relevant cardiovascular complications following pneumonia, which may share common underlying mechanisms, and to increase statistical power.

While a given patient could experience multiple cardiovascular events, only the first occurrence was recorded for analysis.

### 2.4. Data Collection

Past medical history, including previous cardiovascular events, cardiovascular risk factors, and smoking status, was recorded at the time of inclusion. Pneumonia severity, length of hospital stay, ICU admission, Sequential Organ Failure Assessment (SOFA) score, pneumonia severity index score, occurrence of cardiovascular events, and in-hospital mortality were documented during the same hospitalization. As some patients experienced recurrent episodes of pneumonia, only the first recorded episode was considered for analysis.

Patients were then followed for at least 18 months, with follow-up extending from January 2020 to August 2024. The electronic health records of the hospital were reviewed to identify any new cardiovascular events occurring within this period. Patient follow-up data were obtained through review of hospital’s EHR and, when available, from the patients’ primary care physicians. Data were collected by a trained internal medicine physician, and cardiovascular outcomes were based on final diagnoses documented in discharge summaries signed by attending physicians. A large proportion of the patients lived in the region and received regular care at the same hospital, allowing for comprehensive access to their medical records. To complete this, primary care physicians were contacted; however, only 78 (38%) responded and provided information on new cardiovascular events, with non-responses mainly due to refusal to participate, lack of time, or insufficient resources to retrieve the requested data.

### 2.5. Statistical Analysis

We summarized the data using frequencies and percentages for categorical variables. For continuous variables, we used the mean and standard deviation for normally distributed data and the median with interquartile range for non-normally distributed data.

For the primary outcome, we conducted a univariate Cox Model assessing hazards of new cardiovascular events in patients with Frank’s sign compared to those without. Only the first event per patient was considered for the survival analysis. A multivariable Cox proportional hazard model was then performed with adjustment for age, number of cardiovascular risk factors (arterial hypertension, diabetes, dyslipidemia and smoking), pneumonia severity (defined as pneumonia with Pneumonia Severity Indexes—PSI_- ≥ IV or requiring transfer to intensive care unit, as PSI may not fully capture early deterioration or the need for intensive care), and dichotomized previous cardiovascular events including ischemic heart disease, heart failure, peripheral arterial disease, stroke, and atrial fibrillation to address possible cofounders influencing the likelihood of having cardiovascular event. Cardiovascular risk factors were modeled as a count variable to limit model complexity and reduce the risk of overfitting, given the number of events.

The proportional hazards assumption was assessed using Schoenfeld residuals.

In a sensitivity analysis, we constructed a more detailed model using all cardiovascular risk factors as binary variables and binary variables for any history of cardiovascular disease. Statistical significance was established at 0.05.

All statistics were computed using the software R (R Core Team, 2024, Vienna, Austria) V.4.3.0 [13] using the survival package.

## 3. Results

### 3.1. Flowchart of Inclusion

The study flow is presented in Figure 1. A total of 203 patients were included in the final analysis.

### 3.2. Participant Characteristics

Characteristics of the included patients are presented in Table 1. Frank’s sign was present in 71.2% of the included patients. Patients with Frank’s sign were older and had a higher prevalence of cardiovascular risk factors (diabetes, dyslipidemia and hypertension). However, there were no significant differences in comorbidities, or previous cardiovascular events between the groups. Pneumonia Severity Index was greater in the Frank’s sign group, but no differences were observed in terms of length of hospital stay, SOFA score, or ICU admission.

### 3.3. Cardiovascular Events and Predictors

Among the 203 included patients, 103 experienced at least one cardiovascular event during the observation period: 81 in the Frank’s sign group and 22 in patients without the sign. Notably, six observations were deleted due to missingness.

**Figure 2:**
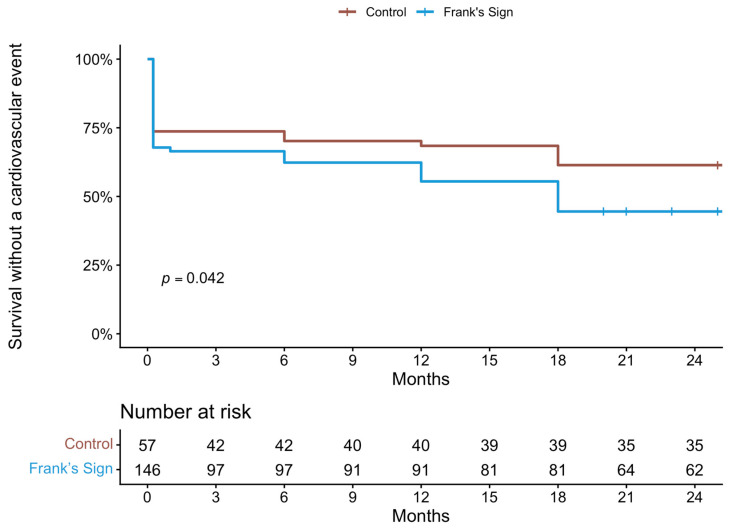
Survival curve of the occurrence of cardiovascular event between the control and Frank’s sign group.

In a univariate Cox proportional hazards analysis, the presence of Frank’s sign was associated with a significantly increased hazard of cardiovascular events compared with patients without the sign (Figure 2) (*p* = 0.042).

In the multivariable Cox proportional hazard model (Table 2), the presence of Frank’s sign was not statistically associated with the occurrence of cardiovascular outcomes after a pneumonia (HR 0.97; 95% CI 0.59–1.59). Only a history of cardiovascular disease and cardiovascular risk factor burden were associated with an increased hazard of subsequent cardiovascular events following pneumonia. The proportional hazard assumption was satisfied for Frank’s sign, whereas pneumonia severity and prior cardiovascular disease showed time-varying effects (Appendix A.1). Results were unchanged in sensitivity analyses using a stratified Cox model for these two variables (Appendix A.2). Notably, age, sex and presence of positive SARS-CoV-2 test did not reach statistical significance in predicting the occurrence of new cardiovascular events.

In a sensitivity analysis, a more detailed cox proportional hazard model using all cardiovascular risk factors as binary variables and binary variables for any history of cardiovascular disease showed similar results and can be found in the Appendix A.3.

## 4. Discussion

To our knowledge, this is the first prospective study to evaluate the occurrence of cardiovascular events following pneumonia in hospitalized patients with Frank’s sign. In unadjusted analysis, Frank’s sign was associated with a higher hazard of subsequent cardiovascular events. However, this association did not persist after adjustment for cardiovascular risk factors and prior cardiovascular disease in both the main and sensitivity analyses. This attenuation suggests that the sign primarily reflects an underlying cardiovascular risk profile rather than independently predicting events. Indeed, patients with Frank’s sign exhibited a higher burden of cardiovascular risk factors and prior events compared with those without the sign, consistent with previously published findings [11]. Frank’s sign could be considered as a simple bedside marker of cardiovascular risk in the acute setting, where traditional screening is often unreliable and diagnostic thresholds are uncertain (e.g., for hypertension, as highlighted by the ongoing SHINE study [14] that will assess the in-hospital hypertension threshold at which patient should receive further outpatient evaluation and treatment). In addition, recognizing this sign should prompt clinicians to communicate potential CV risk to outpatient care providers and ensure appropriate post-discharge screening and preventive measures. Frank’s sign may also have potential utility in other clinical settings, such as preoperative assessment, particularly when prior cardiovascular history is limited, as suggested by a case report in the perioperative setting [15].

Pneumonia itself, like other systemic infections, is well recognized as a trigger of CV events [4,16]. Large prospective studies have shown that patients with pneumonia have a markedly increased risk of CV events compared with controls, particularly within the first 30 days (HR 2.38–4.07), but persisting for up to 10 years (HR 1.86–1.88) [16]. Our findings add to this literature by suggesting that the coexistence of pneumonia and Frank’s sign identifies patients who may be especially vulnerable to such events, in that patients with the sign had significantly more events than the control.

Frank’s sign was associated with a range of different cardiovascular events in our study. Indeed, beyond its known association with coronary heart disease [8,17], Frank’s sign should not be viewed as an exclusive marker of this condition, as its underlying pathophysiology is unlikely to be specific to the coronary circulation. Indeed, recent studies have explored its relationship with other vascular and neurovascular disorders, including ischemic cerebrovascular disease, peripheral arterial disease, and dementia [10,18,19]. Notably, only the first two have demonstrated a positive association with Frank’s sign, with prevalence rates comparable to those observed in coronary heart disease. Taken together, these findings suggest that Frank’s sign may represent a more general marker of systemic vascular pathology rather than a disease-specific indicator. This is further supported by histopathological studies showing arterial myoelastofibrosis, tissue fibrosis, and peripheral nerve degeneration at the base of the crease, consistent with chronic hypoxia-related vascular injury [12], which may drive crease formation in a poorly vascularized lobule and mirror underlying systemic vascular disease.

The inclusion of both viral and bacterial pneumonia, including SARS-CoV-2 infection, introduces heterogeneity that may influence the observed associations. Different etiologies of pneumonia may carry varying risks of subsequent cardiovascular events, which were not specifically accounted for in this study. In our study, a positive SARS-CoV-2 test was not associated with an increased hazard of CVE when adjusting for other covariates. To the best of our knowledge, no other study has evaluated the presence of Frank’s sign during the COVID-19 pandemic. The role of SARS-CoV-2 as a precipitant of cardiovascular events has been previously established [20]. Our findings should however be interpreted with caution given the limited sample size and lack of detailed infection-specific data.

Our study has several strengths. First, its prospective design allowed for the systematic and timely detection of cardiovascular events, thereby minimizing the risk of misclassification and recall bias. The regional hospital setting, which serves a broad catchment area, together with the collection of follow-up data from patients’ general practitioners, improved the completeness of outcome evaluation, although incomplete responses from some practitioners may have led to underestimation. Indeed, outpatient follow-up relied in part on general practitioner responses. With a response rate of 38%, it may have led to incomplete ascertainment of cardiovascular events. Second, the identification of Frank’s sign was conducted in a standardized and rigorous manner. Participating physicians received dedicated training sessions on the recognition of the sign before study initiation, with regular refresher courses held throughout the study period to ensure consistency in assessment. In cases of diagnostic uncertainty, trained study investigators were available to examine patients directly. Third, the inclusion of a well-characterized cohort of hospitalized patients with detailed clinical data allowed for robust adjustment for potential confounders and strengthened the validity of our findings.

Our study has several limitations. First, this study was conducted in a single secondary hospital in Switzerland, within the internal medicine department, which primarily treats older patients with multiple comorbidities and cardiovascular risk factors. This limits the generalizability of our findings to other populations, particularly younger or outpatient cohorts. Further studies are needed to determine whether these findings can be replicated in other hospitals and in outpatient settings. Second, part of the study was conducted during the COVID-19 pandemic, which may limit the generalizability of our findings to non-pandemic periods. Initially, the pandemic could have reduced the patient inclusion rate due to increased clinical workload and strict infection control measures, including isolation protocols. However, this potential limitation is mitigated by the marked increase in pneumonia cases observed during the COVID period. Third, we did not implement a standardized method for documenting or describing Frank’s sign, preventing us from differentiating between unilateral and bilateral presentations. Bilateral or longer and deeper sign has been recently associated with a higher risk of CV events [11]. False interpretation of the sign cannot be entirely ruled out, for example, through the presence of a partial Frank’s sign (a diagonal earlobe crease not extending across the entire lobe) in individuals wearing earrings. This risk was mitigated by repeated demonstrations of the sign to the hospital staff and, when necessary, evaluation by one of the primary investigators. Moreover, this sign, despite its association with CV disease, has also been described in healthy individuals [21] and may sometimes be the results of other processes linked to aging (e.g., solar elastosis) [9,12,22]. Although efforts were made to standardize its assessment through training and repeated demonstrations, interobserver variability was not formally evaluated, and no standardized grading or photographic documentation was used. In our study, compared to the control group, participants presenting with Frank’s sign were significantly older and exhibited a higher prevalence of the classical cardiovascular risk factor. These differences likely contributed to the observed attenuation of the association after adjustment, suggesting that the initial association was largely explained by underlying cardiovascular risk. Moreover, covariates were selected a priori based on clinical relevance and the available literature to capture key sources of confounding. Given the limited number of events, a parsimonious approach was favored to reduce the risk of overfitting. We did not identify sufficient evidence to support the systematic inclusion of additional variables, and their inclusion may have introduced bias. Nevertheless, incomplete measurement of underlying cardiovascular vulnerability may result in residual confounding. Fourth, the validity of cardiovascular events could not be independently verified and relied on third-party physician evaluations from medical records, resulting in possible misclassification bias. Moreover, although pneumonia cases were carefully reviewed, including chest radiography and its interpretation, along with clinical evaluation by the treating physician, some degree of diagnostic uncertainty cannot be excluded. Pneumonia is known to be challenging to diagnose and may be misclassified in routine clinical practice, particularly in older patients with multiple comorbidities. Other conditions, such as cardiogenic pulmonary oedema, may represent potential diagnostic confounders, as they can impair gas exchange and present with clinical and radiographic features similar to pneumonia [23]. Fifth, as patients were screened and referred by treating physicians, inclusion may not have been strictly consecutive, which may introduce selection bias and affect the representativeness of the sample. Finally, although our study was designed prospectively, it is important to note that the follow-up outside the hospital was conducted entirely retrospectively, which may introduce limitations such as missing data and potential biases commonly associated with retrospective data collection.

## 5. Conclusions

In this population of patients hospitalized with pneumonia, Frank’s sign was not independently associated with cardiovascular events. Rather, it appears to reflect underlying cardiovascular risk burden. It should not be interpreted as an independent prognostic marker, but may support the consideration of previously unrecognized cardiovascular risk factors or disease. Traditional risk factors and a history of cardiovascular disease remain the most reliable predictors, although these may not always be readily available during hospitalization. Overall, our findings highlight the value of clinical examination as a simple, non-invasive tool to support cardiovascular risk assessment.

## Figures and Tables

**Table 1:** Characteristics of the patients at inclusion, stratified by demographic variables, CV risk factors, and type and severity of pneumonia. IQR: interquartile range. COVID-19: coronavirus disease 2019 (SARS-CoV-2 infection). * *p*-value < 0.05.

Variables	Control	Frank’s Sign	*p*-Value
Number of patients	57	146	
Age, median [IQR]	71.00 [58.00, 82.00]	76.00 [68.00, 83.00]	0.015 *
Sex, women (%)	25 (43.9)	59 (40.4)	0.772
Body mass index, kg/m^2^ (%)			0.559
<25	30 (52.6)	62 (42.5)	
25–30	17 (29.8)	50 (34.2)	
>30	10 (17.5)	33 (22.6)	
Cardiovascular risk factors			
Hypertension	28 (49.1)	109 (74.7)	0.001 *
Diabetes mellitus	6 (10.5)	43 (29.5)	0.008 *
Dyslipidaemia	6 (10.5)	53 (36.3)	0.001 *
Smoker	28 (49.1)	70 (47.9)	1.0
Cardiovascular diseases			
Ischemic heart disease	8 (14.0)	34 (23.3)	0.204
Acute myocardial infarction	1 (1.8)	9 (6.2)	0.345
Heart failure	11 (19.3)	26 (17.8)	0.964
Atrial fibrillation	10 (17.5)	43 (29.5)	0.119
Peripheral arterial disease	7 (12.3)	27 (18.5)	0.392
History of stroke	6 (10.5)	18 (12.3)	0.908
Other conditions			
Active cancer	8 (14.0)	20 (13.7)	1.0
Obstructive sleep apnea	2 (3.5)	14 (9.6)	0.248
Alcohol use disorder	18 (31.6)	31 (21.2)	0.301
Infectious context			
COVID-19 infection	15 (26.3)	30 (20.5)	0.289
Positive *Legionella* antigen	2 (3.5)	3 (2.1)	0.568
Positive *S. pneumoniae* antigen	9 (15.8)	32 (21.9)	0.399
Severity at admission			
Length of stay, days (median [IQR])	7.00 [6.00, 9.00]	8.00 [6.00, 12.00]	0.165
Pneumonia Severity Index (%)			0.006 *
Class I	3 (5.3)	0 (0.0)	
Class II	15 (26.3)	19 (13.0)	
Class III	10 (17.5)	41 (28.1)	
Class IV	21 (36.8)	66 (45.2)	
Class V	8 (14.0)	20 (13.7)	
SOFA score, median [IQR]	2.00 [1.00, 3.00]	2.00 [1.00, 3.00]	0.846
Admission to intensive care unit (%)	5 (8.8)	12 (8.2)	1.000

**Table 2:** Multivariable Cox proportional hazard model using cardiovascular risk factors as a discrete variable (ranging from zero to four risk factors and including arterial hypertension, diabetes, dyslipidemia and smoking) and a binary variable for any history of cardiovascular disease (including ischemic heart disease, heart failure, peripheral arterial disease, stroke, and atrial fibrillation). COVID-19: coronavirus disease 2019 (SARS-CoV-2 infection).

Variable	HR (95% CI)	*p*-Value
Frank’s sign (present)	0.97 (0.59–1.59)	0.894
Age (per year increase)	1.02 (1.00–1.04)	0.108
Female sex	1.11 (0.68–1.81)	0.670
COVID-19	1.11 (0.62–1.99)	0.738
Severe pneumonia	1.30 (0.78–2.18)	0.319
Cardiovascular risk factors (0–4)	1.32 (1.09–1.59)	0.004
History of cardiovascular disease	2.64 (1.57–4.44)	<0.001

## Data Availability

The data presented in this study are available on reasonable request from the corresponding author.

## Abbreviations

The following abbreviations are used in this manuscript:

FS	Frank’s sign
SOFA	Sequential Organ Failure Assessment score
CVRF	Cardiovascular risk factor
CVE	Cardiovascular event

## Appendix A

### Appendix A.1

Assessment of the proportional hazards assumption using Schoenfeld residuals. The proportional hazards assumption was evaluated for each covariate and globally using Schoenfeld residuals (cox.zph function in the R survival package). *p*-values < 0.05 indicate evidence of non-proportionality (time-varying effects).

**Table A1:** Assessment of proportional hazards using Schoenfeld residuals. COVID-19, coronavirus disease 2019 (SARS-CoV-2 infection).

Variable	Chi-Squared	*p*-Value
Frank’s sign	0.9428247618	0.332
Age	0.5304132663	0.466
Sex	0.6105608768	0.435
COVID-19	0.0002346887	0.988
Severe pneumonia	8.8548822997	0.003
Cardiovascular risk factors (0–4)	0.9421779581	0.332
History of cardiovascular disease	7.3708035863	0.007
GLOBAL	27.3692524755	<0.001

### Appendix A.2

To account for non-proportional hazards in pneumonia severity and prior cardiovascular disease, a stratified Cox proportional hazards model was performed as a sensitivity analysis. The results were consistent with the main analysis.

**Table A2:** Sensitivity analysis using a stratified Cox proportional hazards model. The model was stratified on pneumonia severity and prior cardiovascular disease to account for non-proportional hazards. COVID-19, coronavirus disease 2019 (SARS-CoV-2 infection).

Variable	HR (95% CI)	*p*-Value
Frank’s sign (present)	0.98 (0.59, 1.61)	>0.9
Age (per year increase)	1.02 (1.00, 1.04	0.11
Female sex	1.07 (0.66, 1.73)	0.8
COVID-19	1.06 (0.59, 1.91)	0.8
Cardiovascular risk factors (0-4)	1.30 (1.08, 1.57)	0.006

### Appendix A.3

In the multivariable analysis (Appendix A Table A3), the presence of Frank’s sign was not statistically associated with the occurrence of cardiovascular outcomes after pneumonia (HR 1.07; 95% CI 0.64–1.77). Only a past history of heart failure (HR 7.49; 95% CI 4.26–13.16) or ischemic heart disease (HR 2.06; 95% CI 1.27–3.37) was associated with the hazard of a new cardiovascular event following pneumonia. Notably, age and other traditional cardiovascular risk factors did not reach statistical significance in predicting the occurrence of new cardiovascular events. Assessment of the proportional hazard assumption using Schoenfeld residuals did not show evidence for violation.

**Table A3:** Multivariable cox regression model for cardiovascular event risk. Severe pneumonia refers to pneumonia with a Pneumonia Severity Index ≥ IV or pneumonia that required intensive care transfer.

Variable	HR (95% CI)	*p*-Value
Frank’s sign (present)	1.07 (0.64–1.77)	0.803
Age (per year increase)	1.02 (1.00–1.04)	0.086
Female sex	0.78 (0.48–1.28)	0.329
COVID-19	1.01(0.54–1.87)	0.974
Severe pneumonia	0.84 (0.48–1.47)	0.546
Hypertension	1.18 (0.66–2.10)	0.569
Diabetes	1.44 (0.85–2.42)	0.172
Dyslipidemia	1.13 (0.67–1.90)	0.649
Smoker	0.92 (0.60–1.42)	0.723
Atrial fibrillation	1.14 (0.73–1.80)	0.568
Ischemic heart disease	2.07 (1.27–3.37)	0.004
Heart failure	7.49 (4.26–13.16)	<0.001
Peripheral arterial disease history	0.86 (0.50–1.49)	0.591
Stroke	0.77 (0.41–1.46)	0.427
